# Morphological anomalies in amphibians of the Sierra Madre Occidental and a review of cases reported in Mexico

**DOI:** 10.1007/s10661-025-14157-5

**Published:** 2025-06-05

**Authors:** Héctor Alexis Castro-Bastidas, Marcos Bucio-Pacheco, José Manuel Serrano, David Ramiro Aguillón-Gutiérrez

**Affiliations:** 1 Posgrado en Ciencias Aplicadas al Aprovechamiento de los Recursos Naturales, Centro de Estudios “Justo Sierra” (CEJUS), Badiraguato, 80600 Sinaloa, Mexico; 2 Departamento de Información y Bibliografía Especializada, Facultad de Biología, Universidad Autónoma de Sinaloa, Culiacán, 80013 Sinaloa, Mexico; 3 Departamento El Hombre y su Ambiente, Universidad Autónoma Metropolitana, Unidad Xochimilco, Coyoacán, 04960 Ciudad de Mexico, Mexico; 4 Laboratorio de Bioindicadores, Centro de Investigación y Jardín Etnobiológico, Universidad Autónoma de Coahuila, Unidad Torreón, Viesca, 27480 Coahuila, Mexico

**Keywords:** Amphibian conservation, Bioindicators, Chromatic anomalies, Deformities, Environmental health, Malformations

## Abstract

The data on the incidence and diversity of anomalies in amphibian populations in Mexico are primarily derived from opportunistic observations, which pose challenges for conducting comparisons and monitoring long-term trends. In this study, we document new cases of morphological anomalies in amphibians from the Sierra Madre Occidental and provide a systematic bibliographic review of reported cases across Mexico. During the 2023 rainy season, we conducted surveys in Surutato, Sinaloa, documenting nine cases of morphological anomalies, including the first documented case of dyscoria in the country. The bibliographic review covered 34 sources and identified 31 types of anomalies, with the family Ambystomatidae being the most affected. We observed significant ambiguity in the terminology used across reports, which hinders data comparability. To address this issue, we propose promoting a standardized approach to the description and classification of anomalies. Based on the available data, we estimate a national mean anomaly frequency of 18.43% in amphibians, which can serve as a preliminary benchmark for future studies. The most commonly identified causes of anomalies include agrochemical pollution and parasitism; however, in 37% of the cases, no specific cause could be determined. These findings underscore the importance of standardizing anomaly reports, as they can serve as valuable early indicators of environmental degradation.

## Introduction

Ecosystem health is commonly assessed using bioindicators that exhibit physical, chemical, or behavioral responses to environmental stressors (Hee, 1993). Commonly used bioindicators in environmental assessment studies include animals (e.g., fish, birds, macroinvertebrates), plants (e.g., tree growth rings, mosses), fungi (e.g., mycorrhizal fungi, molds, yeasts), lichens, algae (e.g., diatoms, green algae), and other microorganisms (e.g., bacteria, protozoa) due to their sensitivity to environmental disturbances (Bonanno et al., 2020; Gerhardt, 2002; Hinojosa-Garro et al., 2020; Prazeres et al., 2020). Proper interpretation of bioindicators is crucial for obtaining accurate assessments of ecosystem health and for implementing effective conservation strategies, as bioindicators provide qualitative or quantitative information on the effects of environmental stressors (Gerhardt, 2002).

Bioindicators are classified into accumulation indicators, which retain pollutants without exhibiting visible changes in their metabolism, and response indicators, which display symptoms of environmental stress upon absorbing harmful substances (Hayes et al., 2006; Witt, 1996). Amphibians such as *Bufotes viridis* and *Pseudacris regilla* exhibit anomalies such as polymely (extra limbs) and ectrodactyly (digit loss) when exposed to pollutants (e.g., heavy metals, pesticides, hydrocarbons) or radiation. Additionally, trematode infections (*Ribeiroia ondatrae*) disrupt limb development, causing deformities and skin fusion (Henle et al., 2017a; Johnson et al., 1999, 2003; Rohr et al., 2003). These physiological and morphological disruptions highlight the sensitivity of amphibians to environmental stressors. Monitoring bioindicator species helps identify and evaluate environmental impacts, providing a deeper understanding of ecosystem health and facilitating the implementation of effective environmental management measures (Walker et al., 2012).

Most amphibians breathe through their thin, permeable skin, which not only facilitates oxygen absorption (Heatwole, 1994; Tattersall, 2007) but also allows certain contaminants (e.g., herbicides such as atrazine, paraquat, and glyphosate) to enter their bloodstream (Quaranta et al., 2009). This high permeability facilitates the absorption of toxic substances, which can induce morphological or chromatic anomalies commonly associated with exposure to chemical pollutants or environmental disturbances (Van Meter et al., 2018; Welsh & Ollivier, 1998). Amphibian morphological anomalies are typically categorized into two types: malformations, which are congenital structural alterations, and deformities, which are acquired during or after development (Meteyer, 2000). Both types may arise from the influence of contaminants or environmental disturbances (Henle et al., 2017a; Johnson et al., 2010). In contrast, chromatic anomalies, characterized by unusual coloration patterns, do not necessarily involve structural deviations and may reflect physiological responses to various environmental stressors (Henle et al., 2017b).

The categorization of amphibian anomalies serves as an effective indicator of environmental health, as these anomalies are often associated with developmental errors induced by specific external factors, such as contaminants (Blaustein & Johnson, 2003; Severtsova et al., 2012; Vershinin, 1989). However, not all anomalies have a direct environmental cause (Serrano, 2011). In certain cases, anomalies may result from genetic mutations unrelated to environmental factors (Hamburger, 1935; Krotoski et al., 1985; Mable & Rye, 1992; Tolledo & Toledo, 2015). These mutations can occur spontaneously and represent part of the natural genetic variation within a population (Hitchings & Beebee, 1997, 1998; Weyrauch & Grubb, 2006).

Differentiating between genetic and environmentally induced anomalies requires a multidisciplinary approach. Experimental studies, such as controlled exposure to pollutants in laboratory settings, can isolate teratogenic effects (Johnson et al., 2003; Rohr et al., 2003). Field-based correlations between anomaly prevalence and contaminant levels (e.g., heavy metals, pesticides) further support environmental causation (Henle et al., 2017a; Vershinin, 2002). Conversely, genetic anomalies are identified through pedigree analyses, heritability tests, or the persistence of anomalies in captive-bred populations under contaminant-free conditions (Hamburger, 1935; Kawamura & Nishioka, 1978). Molecular tools, such as sequencing candidate genes or genome-wide association studies, are increasingly used to pinpoint mutations (Nishioka & Ueda, 1985; Tolledo & Toledo, 2015). However, disentangling these causes is complex, as genetic predispositions may amplify susceptibility to environmental stressors (Henle et al., 2017b; Kiesecker, 2002).

The identification and analysis of genetic anomalies are crucial for distinguishing between anomalies caused by pollution or habitat degradation and those that represent normal biological processes. This differentiation is essential for accurately understanding the factors affecting amphibian populations and environmental health. Moreover, identifying these anomalies provides early warning signs of habitat degradation and pollutant presence (Rapport, 1992; Rapport & Regier, 1995), facilitating the implementation of appropriate management measures.

Most detailed studies on the underlying causes of amphibian anomalies have been conducted in the United States and Europe (Henle et al., 2017a). In contrast, in Latin America, the increase in reports over the past decade has primarily focused on case descriptions, with limited analysis of specific causes (Beltrán-Álvarez, 2023). In Mexico, reports of morphological anomalies have risen in recent years (Cruz-Pérez et al., 2009; Soto-Rojas et al., 2017; Venerozo-Tlazalo et al., 2022). Although Venerozo-Tlazalo et al. (2022) provided an updated list of morphological anomalies observed in amphibians from Mexico, they excluded chromatic anomalies, which may also hold biological relevance. Chromatic anomalies, such as albinism, melanism, and erythrism, can affect camouflage, thermoregulation, and predator avoidance, thereby influencing individual survival and fitness (Henle et al., 2017a; Vershinin, 2002). This exclusion limits the ability to fully associate environmental effects, such as pollution, with abnormal development in amphibians, resulting in an incomplete perspective on the threats they face.

In this context, we present new records of morphological anomalies in amphibians from a locality in the Sierra Madre Occidental (SMO) and review previously reported cases of anomalies in Mexico, including both morphological and chromatic anomalies. By analyzing the incidence, distribution, and potential causes of these anomalies in Mexico, we aim to deepen the understanding of amphibian population health and propose guidelines for future research to better address the factors impacting their well-being.

## Methods

### Study area

**Fig. 1:**
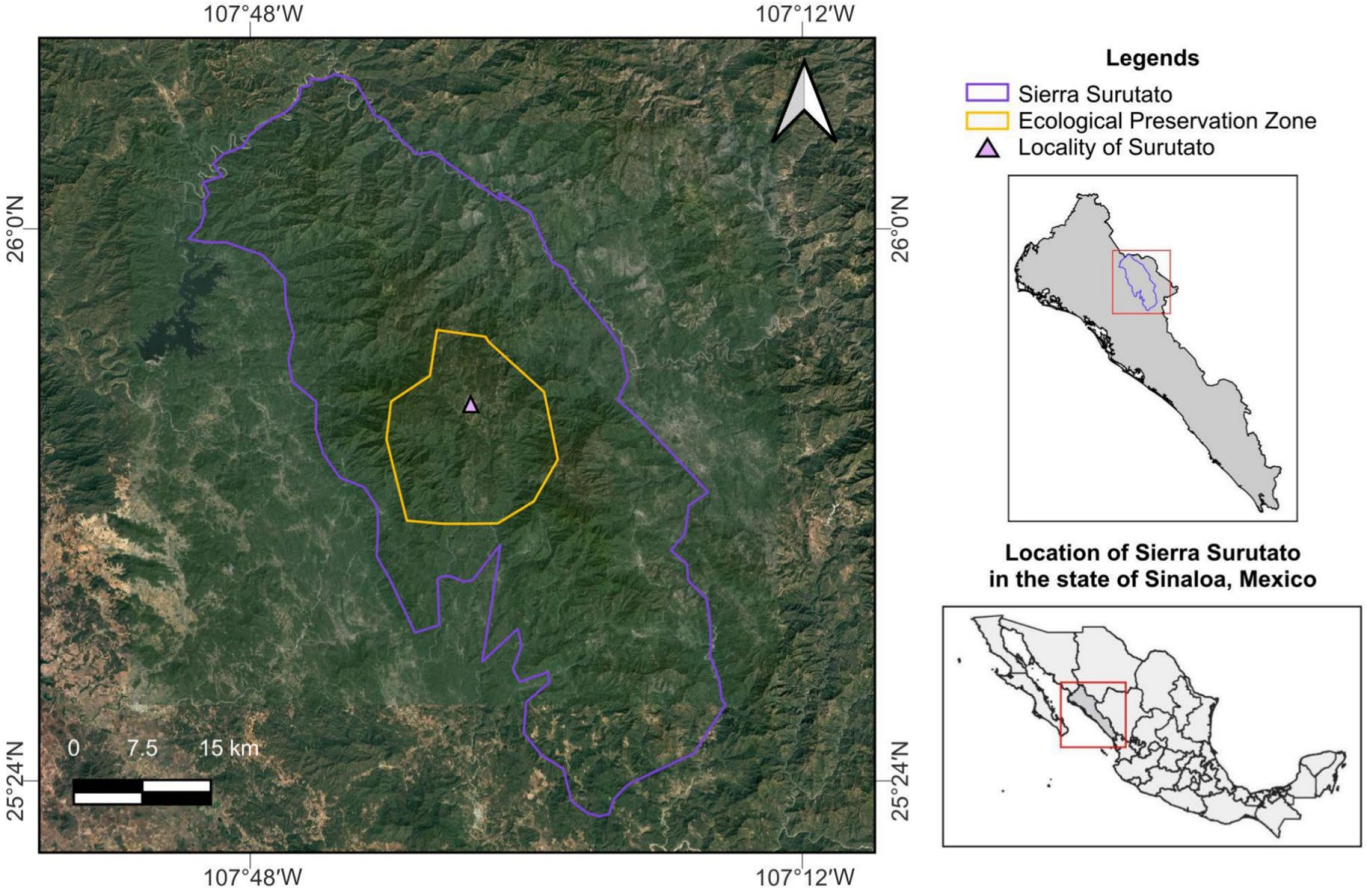
Study site where morphological anomalies reported in this work were observed

The mountainous region of Sierra Surutato spans 2376 km^2^ in the northeastern Sinaloa, Mexico, bordering the states of Chihuahua and Durango within the SMO. This area encompasses the Surutato Ecological Preservation Zone (25°47′08″ N, 107°33′20″ W) in the municipality of Badiraguato, with elevations ranging from 1500 to 1800 m.a.s.l. and predominantly covered by pine and oak forests (Fig. 1). This region has a subhumid temperate climate with summer rains (CICESE, 2020), characterized by an annual average temperature of 24.8 °C. In winter, temperatures occasionally drop to − 2 °C, accompanied by frost events. Annual precipitation averages 1156.9 mm, with most rainfall occurring during the rainy season (June to September), while the dry season extends from October to May.

### Fieldwork for identification of anomalies

We conducted amphibian monitoring in Surutato from July to October 2023 as part of a study investigating ENSO-related patterns of amphibian diversity. We complemented the sampling with additional non-systematic visits in September, during the rainy season of the same year. Sampling efforts consisted of three campaigns, each lasting 3 to 5 days and spaced 30–32 days apart. Each campaign involved three researchers, totaling 89.75 person-hours of effort. We surveyed three sites with distinct environmental characteristics: pine forest, oak forest, and a semi-urban area. Searches focused on microhabitats such as substrate, streams, water bodies, rocks, and logs, using active visual encounter surveys during both day and night (Heyer et al., 1994). For captured individuals, we recorded snout-vent length (SVL) and body weight. We identified species using previous inventories and descriptive documents from the region (Hardy & McDiarmid, 1969; McDiarmid et al., 1976). To ensure strict biosecurity, we adhered to established protocols, including the use of individual gloves for each captured specimen and the disinfection of measuring tools with 70% alcohol (Phillott et al., 2010).

As part of our documentation process, individuals captured at night were temporarily retained until daylight for optimal photography, ensuring accurate identification and preventing duplicate counts in subsequent samplings. No additional examinations were performed after documentation, and all individuals were released at the same location where they were captured. Anomalies were identified and verified from these photographs using the criteria described by Henle et al. (2017b) and Ross and Plummer (2022) for ophthalmological cases. In this study, we use the term “anomalies” as a general descriptor for any alteration, reserving the specific terms “malformations” and “‘deformities” for structural contexts.

### Systematic literature review

Our approach was based on the standardized Preferred Reporting Items for Systematic Reviews and Meta-Analyses (PRISMA) method for document retrieval and analysis (Moher et al., 2015). We conducted an extensive review of the literature published up to April 2024 on amphibian anomalies in Mexico, focusing on morphological and chromatic anomalies as well as deformities. Although deformities are not congenital, we included them in this review due to their frequent conflation with morphological anomalies in scientific literature and their biological significance (Henle et al., 2017b). To ensure conceptual consistency, we standardized the definitions of anomalies reported in Mexico with the terminology proposed by Henle et al. (2017b).

Our literature search involved multiple scientific databases, including Web of Science, SCOPUS, Semantic Scholar, Google Scholar, Scielo, Latindex, and REDALyC. We used the keywords “malformation,” “deformity,” “abnormality,” “anomalies,” “amphibians,” “amphibia,” and “Mexico,” combined with the Boolean operators AND and OR. The search profile was “(malformation OR deformity OR abnormality OR anomalies) AND (amphibian OR amphibia) AND Mexico” including searches in Spanish. For Spanish-language databases like Latindex and REDALyC, we refined the search to focus on Mexico and specific disciplines, such as biology, herpetology, and veterinary medicine.

To ensure comprehensive coverage, we reviewed articles published in specialized journals, including *Journal of Herpetology*, *Herpetology Notes*, *Herpetological Review*, *Reptiles & Amphibians*, *Revista Latinoamericana de Herpetología*, and *Cuadernos de Herpetología*, among others (see Table 3 in the Appendix for sources). Additionally, we employed snowballing or cascading searches (Wohlin et al., 2022) by examining the references cited in retrieved articles. We filtered the results to exclude articles that mentioned keywords without presenting specific cases. Our review included studies on natural populations, captive populations, and laboratory animals, but excluded those involving organisms experimentally exposed to contaminants (e.g., Aguillón-Gutiérrez & Ramírez-Bautista, 2015).

### Analysis of bibliographic information

We quantified the annual scientific production and, incorporating the new records from this study, described and quantified the types and numbers of morphological and chromatic anomalies. We also categorized the cases by amphibian family, developmental stage, and geographical distribution within Mexico. Additionally, we examined potential causes of the anomalies, emphasizing those linked to the biological and environmental context of each case as described by the authors.

### Frequency analysis of anomalies in Surutato and Mexico

We calculated the frequency of morphological anomalies observed during amphibian monitoring in Surutato as the percentage of individuals sampled that exhibited such anomalies (Henle et al., 2017a). Additionally, we estimated the national mean frequency of amphibian anomalies in Mexico, addressing a limitation in over 50% of the reports, which either lack sample size information or are based on incidental encounters. For instance, in Surutato, two cases could not be included in frequency calculations due to their incidental nature, where no sample size was recorded, restricting result interpretation (see the “Results” section for details). The national mean frequency (NM) was calculated using the formula: 

$$NM=(Na/Ns)\times100$$

where:

NM is the national mean frequency of anomalies.

*Na* is the number of individuals reported with anomalies.

*Ns* is the total number of individuals sampled.

## Results

### Systematic review of studies on amphibian anomalies in Mexico

**Fig. 2:**
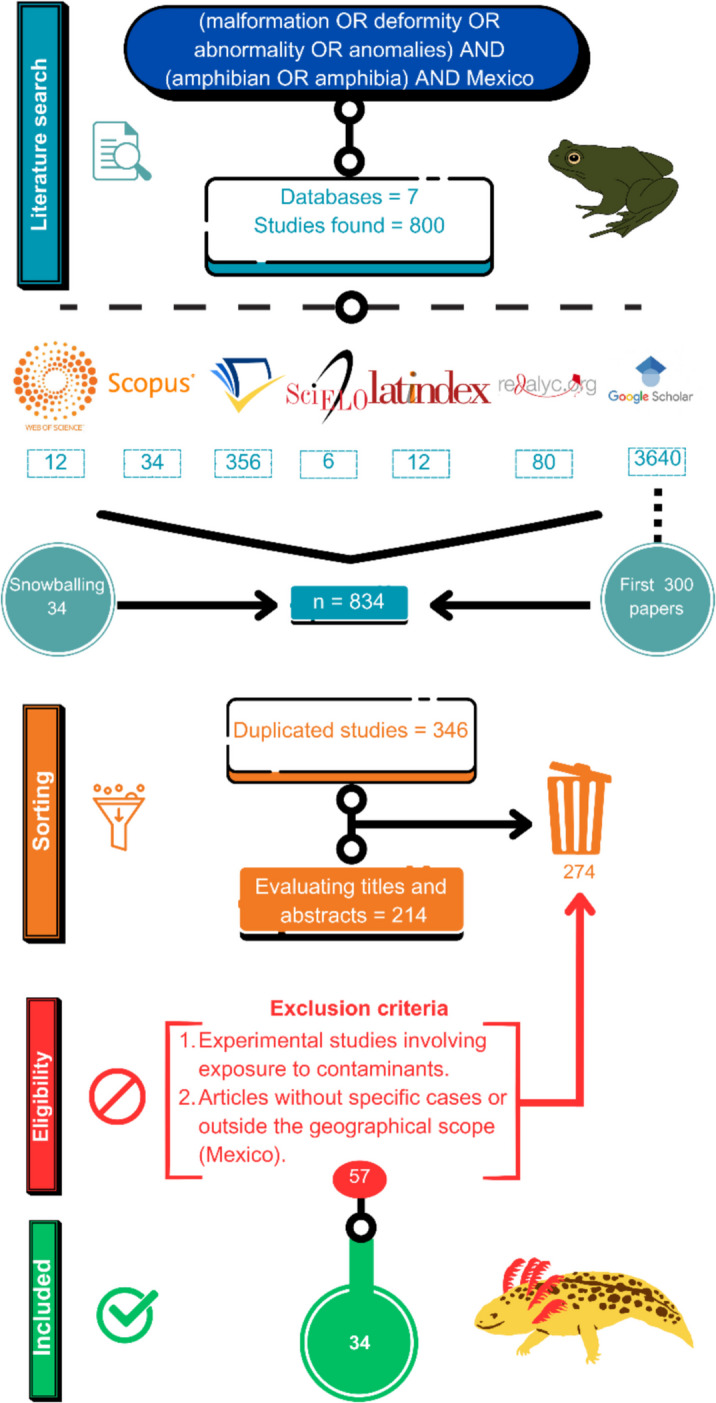
Flowchart illustrating the selection process of articles on amphibian anomalies in Mexico, following PRISMA criteria (Moher et al., 2015)

The flowchart illustrates the systematic process used to select studies on amphibian anomalies in Mexico (Fig. 2). An initial search across seven databases identified 834 documents. After removing 346 duplicates and excluding 274 documents that did not meet the predefined eligibility criteria (see the “Methods” section), the remaining articles underwent a thorough evaluation. Ultimately, 34 studies met all methodological and thematic requirements and were included in the final analysis.

### New records of morphological anomalies in amphibians from Surutato

**Fig. 3:**
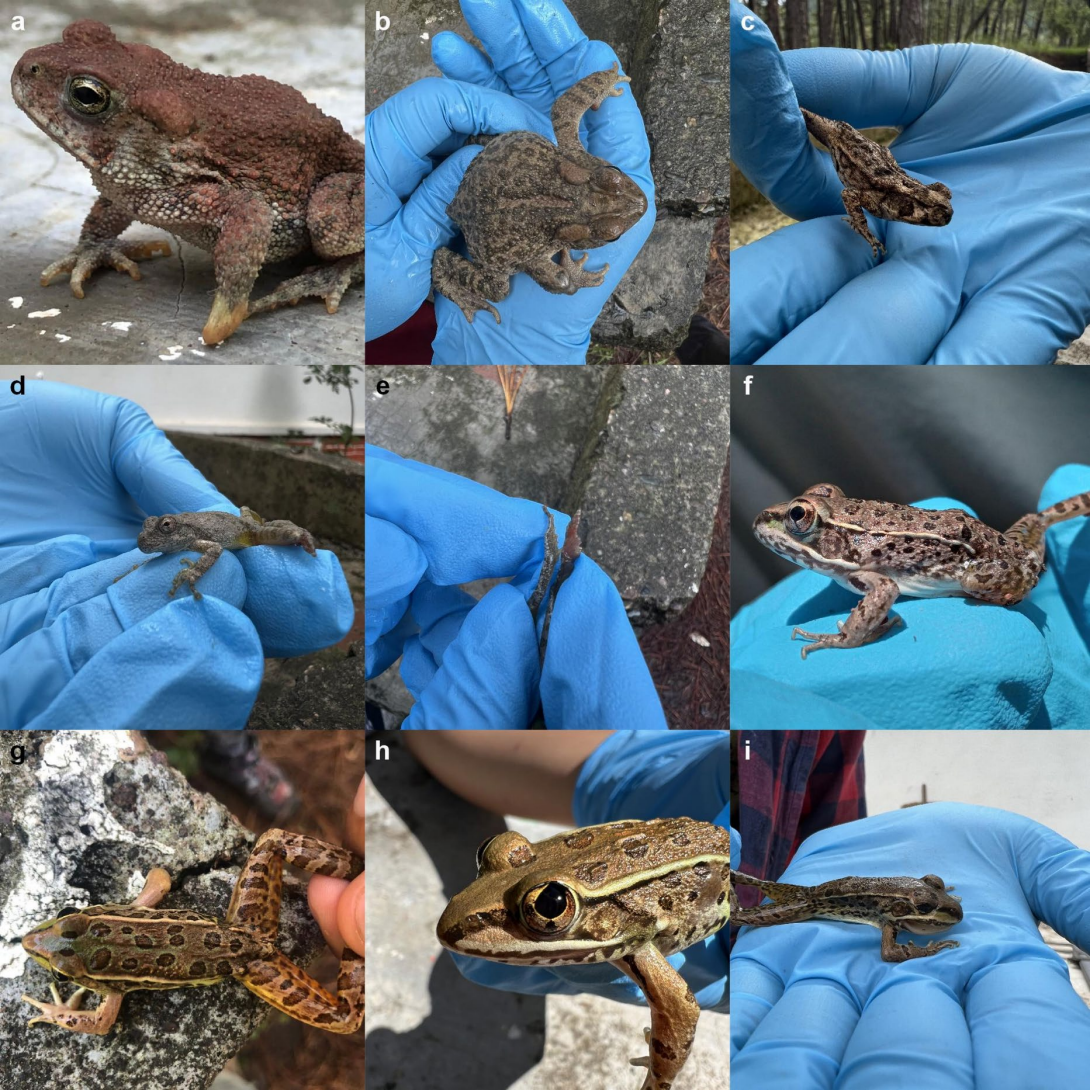
*Incilius mccoyi*: **a** deformity and **b** limb protrusions. *Rhinella horribilis*: **c** anophthalmia. *Dryophytes arenicolor*: **d** and **e** brachydactyly. *Lithobates magnaocularis*: **f** femoral ectromely, **g** radio-ulna ectromely, **h** dyscoria, and **i** microphthalmia with mandibular brachygnathia. Photographs by MBP (**a** and **g**), Heleana Velarde Urías (**b**, **c**, **d**, **e**, **h**, and **i**), and José David Jacobo González (**f**)

We documented nine cases of morphological anomalies in amphibians from the Sierra Surutato, Sinaloa, Mexico, based on both systematic and non-systematic surveys conducted during the 2023 rainy season (Fig. 3). Two of these cases are potential deformities (definitions of anomalies can be reviewed in Table 4 in the Appendix). Details of each case are provided in Table 1.

**Table 1:** Cases of morphological anomalies in Surutato, Sinaloa, Mexico

Species	Date	Hour	Life stage/sex	SVL (mm)	Habitat	Coordinate	Elevation (m a.s.l.)	Anomalies	Figure 2
*Incilius mccoyi*	24 Sep 2023	20:00	Juvenile	39	Pine forest near cabins	25°49′44.94″ N, 107°33′53.53″ W	1527	Deformity	a
	5 Oct 2023	21:48	Female	64	Pine forest near cabins	25°49′44.94″ N, 107°33′53.53″ W	1527	Deformity	b
*Rhinella horribilis*	29 Aug 2023	19:00	Juvenile	56	Near a stream in oak-dominated vegetation	25°49′48.19″ N, 107°33′53.07″ W	1537	Anophthalmia	c
*Dryophytes arenicolor*	8 Oct 2023	10:05	Juvenile	34	On a rock in a stream within oak-dominated vegetation	25°49′48.89″ N, 107°33′52.31″ W	1540	Brachydactyly	d–e
*Lithobates magnaocularis*	30 Jul 2023	20:07	Juvenile	43	Pine forest near cabins	25°49′33.91″ N, 107°33′57.89″ W	1488	Femoral ectromely	f
	28 Sep 2023	21:00	Male	58	Pine forest near cabins	25°49′33.91″ N, 107°33′57.89″ W	1488	Radio-ulna ectromely	g
	5 Oct 2023	22:36	Male	48	Pine forest near cabins	25°49′57.69″ N, 107°33′58.13″ W	1530	Dyscoria	h
	6 Oct 2023	21:00	Juvenile	25	Disturbed pine-oak forest	25°48′55.82″ N, 107°33′42.97″ W	1457	Microphthalmia and inferior brachygnathia	i

### Occurrence frequency and national mean of anomalies

Data from our literature review and newly documented records indicate that 345 out of 1872 individuals sampled nationwide exhibited anomalies, resulting in a national mean frequency of 18.43% (Supplementary Material). For the species documented in Surutato, we calculated the frequency of morphological anomalies for five species (Table 2). However, in two cases based on incidental encounters, frequency calculations were not feasible due to the absence of a defined sample size (e.g., cases in Fig. 3a and g). Most species in Surutato displayed a frequency below the national mean, except *I. mccoyi*, which exhibited a notably high frequency of 50% (Table 2). Despite this, the small sample size for this species precludes definitive conclusions. As two cases lacked defined sample sizes, we refrained from calculating a regional mean, as incomplete data limit result interpretation.

**Table 2:** Frequency of morphological anomalies observed during the sampling periods of this study. In some cases, calculations could not be performed because the anomalies were not part of the systematic monitoring

Type of sampling	Species	Type of anomalies	Frequency^a^	N	This study (Fig. 2)
Non-systematic	*Incilius mccoyi*	Deformity	—	—	a
	*Lithobates maganaocularis*	Radio-ulna ectromely	—	—	g
Systematic	*Incilius mccoyi*	Deformity	50%	2	b
	*Rhinella horribilis*	Anophthalmia	6.66%	15	c
	*Dryophytes arenicolor*	Brachydactyly	20%	5	d and e
	*Lithobates maganaocularis*	Femoral ectromely	6.66%	15	f
		Dyscoria, microphthalmia, and brachygnathia	1.42%	140	h and I

^a^The national mean calculated for Mexico is 18.43 with a standard deviation of 0.00896

### Amphibian anomalies in Mexico

Our literature review identified 34 documents describing 31 types of amphibian anomalies in Mexico, encompassing 90 reported cases. A total of 34 species with recorded anomalies were identified, including *I. mccoyi* and *L. magnaocularis*, which represent novel records of anomalies, with all cases documented as first records (Table 1). Additionally, a temporal analysis of publications reporting amphibian anomalies in Mexico reveals a notable increase beginning in 2021, peaking in 2022 and 2023, with seven documents published each year. Prior to 2021, the number of publications was significantly lower, ranging from zero to two per year (Supplementary Material).

**Fig. 4:**
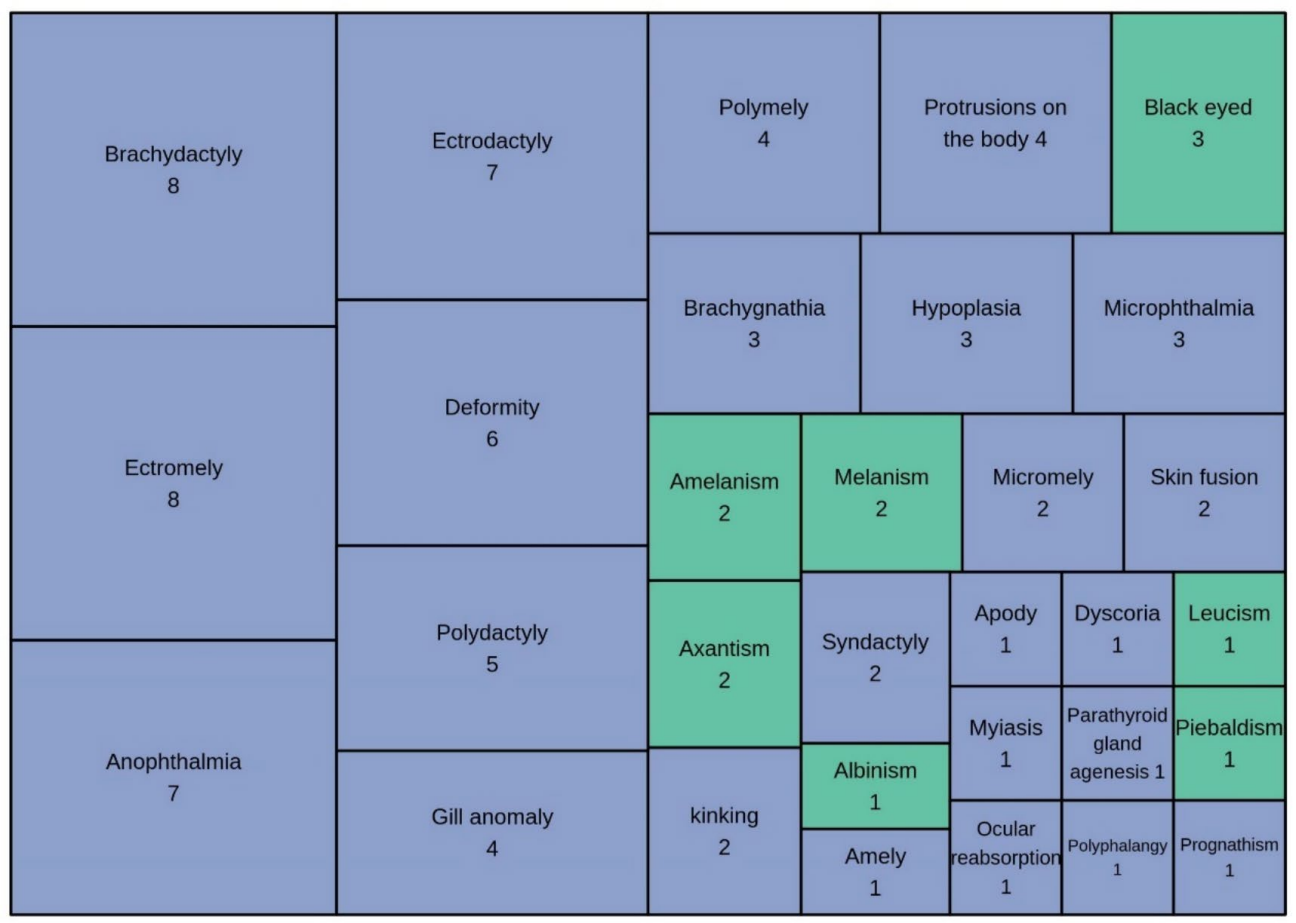
Types of anomalies reported in amphibians in Mexico. Green areas represent chromatic anomalies, while blue areas indicate morphological anomalies (malformations and deformities)

We identified seven chromatic anomalies (*n* = 12) and 24 morphological anomalies (*n* = 78; Fig. 4). The most frequent malformation was ectromely (9%, *n* = 8), followed by anophthalmia, brachydactyly, and ectrodactyly (8%, *n* = 7). Rare anomalies such as agenesis of the parotoid gland, amely, apody, dyscoria, myiasis, polydactyly, prognathism, and ocular reabsorption were reported only once. Definitions are provided in Table 4 of the Appendix.

### Frequency of anomalies by family and development stages

**Fig. 5:**
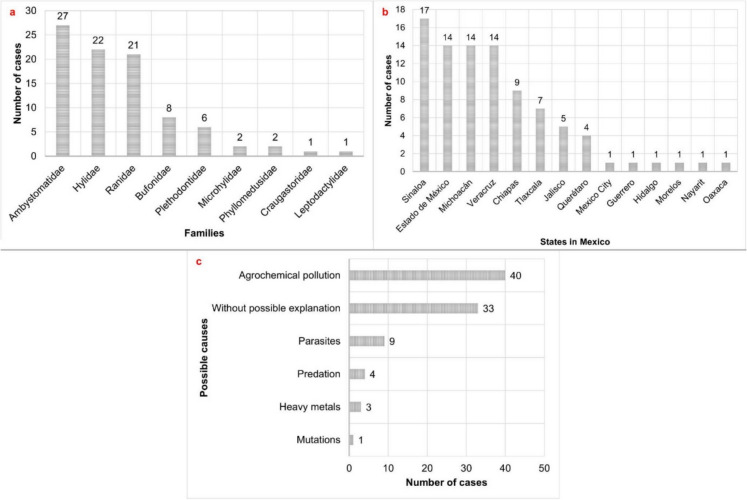
Number of chromatic and morphological anomaly cases in amphibians reported in Mexico by **a** families, **b** states, and **c** suggested causes

In Mexico, the amphibian families with the highest frequency of anomalies are Ambystomatidae (30%, *n* = 27), Hylidae (24%, *n* = 22), and Ranidae (23%, *n* = 21). In contrast, Craugastoridae and Leptodactylidae exhibit the lowest frequency, with only one case reported for each (Table 3 of the Appendix; Fig. 5a). Regarding developmental stages, adults account for the majority of reported cases (43%, *n* = 39), followed by juveniles (24%, *n* = 22) and larvae (5%, *n* = 5). Additionally, 27% of cases (*n* = 24) did not specify the developmental stage of the individuals.

### Geographic distribution and causes of anomalies

Sinaloa reported the highest frequency of anomalies (19%, *n* = 17), followed by the Estado de México, Michoacán, and Veracruz (15%, *n* = 14). Notably, 56% of Mexican states (*n* = 18) have not documented any cases (Fig. 5b).

Possible causes of anomalies included agrochemical pollution (44%, *n* = 40), parasites (10%, *n* = 9), and other factors such as predation, heavy metals, and mutations (Fig. 5c). For 37% of cases (*n* = 33), no specific cause was identified.

## Discussion

The significance of this study lies in the integration of field data from Sinaloa with a comprehensive systematic review of the literature on amphibian anomalies in Mexico. This combined approach provides a more complete understanding of the frequency and diversity of these anomalies. Compared to the work of Venerozo-Tlazalo et al. (2022), our study significantly expands the geographic and taxonomic scope of documented cases, adding 20 new species records and reporting anomalies in eight additional states. Notably, we document the first record of dyscoria in Mexico, an alteration in the shape or arrangement of the pupils (Ross & Plummer, 2022). These observations not only expand the catalog of known anomalies but also underscore the need for ongoing monitoring to better understand the environmental and biological factors contributing to their occurrence.

### The case of morphological anomalies in the Surutato region

The recurrence of sampling at the same sites allows us to observe that the frequency of morphological anomalies in amphibians from Surutato is not consistent across species (Table 2). *Rhinella horribilis* exhibited an anomaly frequency of 6.66%, while *L. magnaocularis* showed variable frequencies across distinct anomaly cases: 6.66% in one case, 1.42% in another, and an incalculable frequency in the third. In contrast, *D. arenicolor* and *I. mccoyi* exhibited much higher frequencies, at 20% and 50%, respectively. Although *I. mccoyi* showed a 50% anomaly frequency, considerably higher than the estimated national mean (18.43%), this result should be interpreted with caution due to the small sample size (only two individuals). Given the limited sample sizes in some cases, a specific average for Surutato was not calculated. However, the available data suggest that the anomaly frequency in this region may be relatively low compared to the national mean. This conclusion should be interpreted cautiously, as overall reporting remains inconsistent.

Previous studies indicate that the expected anomaly rate in amphibian populations is typically low, ranging from 0 to 5% in healthy populations (Blaustein & Johnson, 2003; Ouellet, 2000). These low rates are likely influenced by biological factors, such as high mortality of anomalous individuals and natural selection against severe anomalies, as well as methodological biases, including under-detection of mild anomalies or small sample sizes (Reeves et al., 2013). Reeves et al. (2013) suggest that anomalies commonly occur in approximately 0 to 2% of individuals in wild populations. Similarly, Blaustein and Johnson (2003) assert that no more than 5% of a healthy population should exhibit anomalies to remain within a “normal” range.

Our study is pioneering in this field, as more than 50% of the reports lack information on sample size (Supplementary Material), contrasting with studies that analyze sample sizes exceeding 5000 individuals across various amphibian species (Henle et al., 2017a). While some populations exhibit significantly higher anomaly rates, with some cases exceeding 15% (e.g., Severtsova et al., 2012; Vershinin, 1989), these instances are exceptions and often reflect underlying ecological disturbances. Therefore, although our data provide valuable insights, they should be interpreted with caution. The national mean may become more accurate as future research incorporates larger sample sizes.

In Surutato, Sinaloa, a more detailed analysis is needed to identify the factors driving these anomalies. Accurate identification of the causes is essential to determine whether the observed frequencies reflect natural phenomena or adverse environmental pressures. Future studies should aim to expand sample sizes and implement standardized protocols to explore the relationship between environmental variables and anomaly prevalence.

### Increase in reports of amphibian anomalies in Mexico

The analysis of scientific production on morphological and chromatic anomalies in amphibians from Mexico reveals an upward trend in their documentation, especially since 2016 (Supplementary Material). Notably, 2022 and 2023 had the highest number of publications, with seven studies each year. This trend reflects growing interest and effort from the scientific community to investigate these phenomena. However, there remains a need to develop specific protocols that facilitate the search, recording, and reporting of anomalies in a more coordinated and systematic manner, as many current records are incidental (e.g., Carmona-Zamora et al., 2020; Cruz-Pérez et al., 2009; Del Moral-Flores et al., 2022).

This increased interest may stem from a heightened awareness of biodiversity and environmental health, as evidenced by global efforts to document and conserve amphibian populations in response to widespread environmental changes (Rohwer et al., 2022). Additionally, the growing number of publications on this topic reflects a broader trend in ecological research, where amphibian anomalies are increasingly recognized as indicators of environmental stress and ecosystem health (Ferrante & Fearnside, 2020; Gonçalves et al., 2019; Zhelev et al., 2018). Although many reports are preliminary, they make a significant contribution to understanding the incidence of amphibian anomalies in Mexico by expanding the catalog of documented cases. Notably, most reports have been led by a small group of researchers specializing in amphibian ecology, facilitating long-term trend monitoring in our study. However, encouraging authors to provide robust conclusions is essential for identifying patterns and exploring the origins of these anomalies.

To improve the detection and documentation of anomalies in amphibians, it is essential to adopt standardized methodologies that enable the collection of more accurate and comparable data. This includes leveraging scientific collections to uncover historical patterns of anomalies and establish temporal baselines (e.g., Sanders et al., 2023). The implementation of detailed field guides, the use of photographic documentation, and the creation of centralized databases would facilitate information sharing and enable large-scale pattern analysis (e.g., Hedley, 2014; Johnson et al., 2000; Meteyer, 2000). Furthermore, training researchers and engaging citizen scientists could improve the quality of records, thereby strengthening conservation efforts for these organisms (e.g., Mason et al., 2024).

### Types and frequency of anomalies in amphibians in Mexico

Among the most prevalent morphological anomalies documented in amphibians in Mexico are ectromely, ectrodactyly, and brachydactyly (Fig. 4). These alterations are primarily associated with exposure to chemical pollutants, such as pesticides and herbicides in the case of ectromely (Grosse & Bauch, 1988; Mizgireuv et al., 1984; Reeves et al., 2013). Ectrodactyly and brachydactyly have been linked to various factors, including trauma, parasitic infections, radiation exposure, and unknown causes (Bezman-Moseyko et al., 2014; Gray et al., 2002; Johnson & Hartson, 2009; Tyler, 1989).

Another recurrent anomaly, anophthalmia, may be caused by chemical contaminants during embryonic development (Fort et al., 2006b; Reeves et al., 2008), predation during larval stages (Ballengée & Sessions, 2009), inbreeding (Tolledo & Toledo, 2015), hybridization (Mable & Rye, 1992), and genetic factors (Krotoski et al., 1985). However, the precise cause of ocular globe reduction or absence in natural amphibian populations remains unclear (Henle et al., 2017a).

Less common morphological anomalies, such as polydactyly, syndactyly, and prognathism, require more in-depth studies to identify their causes. For instance, polydactyly may be linked to parasitic infections (Johnson et al., 2003, 2006; Svinin et al., 2020), while syndactyly has associations with wastewater contamination, agricultural activities (Bacon et al., 2006; Fort et al., 2006a), and genetic predispositions (Hamburger, 1935). Jaw malformations, such as prognathism, may be related to pesticide and radiation exposure (Tyler, 1989).

Regarding chromatic anomalies, such as albinism and axanthism, these are typically caused by mutations affecting skin and eye pigmentation (Bagnara et al., 1978; Frost et al., 1984). Chemical residues in aquatic habitats, such as ammonium nitrate and insecticides, have also been implicated (Pandey & Tomar, 1985; Park et al., 2010; Scaia et al., 2019). Elevated radiation levels could induce these mutations (Labrousse, 1967). Although many chromatic anomalies are attributed to genetic factors (Henle et al., 2017a), the incidence of mutations as a cause of these anomalies may be underestimated. In Mexico, a lack of genetic studies focused on amphibian species with high anomaly frequencies (Aguillón-Gutiérrez, 2018) limits understanding of hereditary influences in local populations.

### Patterns of anomalies by developmental stages and amphibian families in Mexico

Anomaly frequency patterns in amphibians vary considerably across developmental stages. Adults and juveniles exhibit a higher apparent frequency of anomalies due to their larger size and the ease of observation. In contrast, larvae show lower frequencies, possibly due to their shorter lifespan and the lack of targeted studies (Aguillón-Gutiérrez, 2018). This trend suggests that many larval individuals may not complete metamorphosis, indicating that the anomalies observed in adults are influenced not only by congenital factors but also by epigenetic processes (Dournon, 1983; Kovalenko, 2000; Vershinin, 2004).

Differences among families also reveal distinctive patterns in anomaly frequency in Mexico, particularly within the families Ambystomatidae, Hylidae, and Ranidae (Fig. 5a). Salamanders of the genus *Ambystoma* (e.g., axolotls) exhibit notably high anomaly rates, with *Ambystoma ordinarium* being one of the most affected species (Table 3 of the Appendix; Soto-Rojas et al., 2017). The vulnerability of Ambystomatidae is associated with their reliance on aquatic habitats in degraded water bodies, biological traits like permeable skin that enhance toxin absorption (e.g., Quaranta et al., 2009; Van Meter et al., 2018), and prolonged larval stages that increase exposure to pollutants (Wake & Vredenburg, 2008). Likewise, Hylidae, the most diverse anuran family in Mexico (with 99 species, Ramírez-Bautista et al., 2023), exhibit a high susceptibility to anomalies despite their adaptability to disturbed environments (Camarena-Hernández et al., 2023), likely due to chronic exposure to agrochemicals and pollutants from urbanization. Meanwhile, Ranidae species, which frequently inhabit shallow water bodies near agricultural zones, face heightened risks from pesticide contamination and habitat fragmentation (Hayes et al., 2006). These patterns may, in part, reflect uneven research efforts, as Ambystomatidae in accessible lowland ponds (e.g., central Mexico) have been more intensively studied than taxa in remote or underfunded regions, potentially inflating reported anomaly rates.

### Geographical distribution of amphibian anomalies in Mexico

An analysis of amphibian anomaly frequency across Mexico reveals significant variation among states (Fig. 5b). Sinaloa emerges as a hotspot for reported cases, particularly in the areas of Surutato and El Palmito (Castro-Bastidas et al., 2022a, 2024; this study). While herpetofaunal research in Sinaloa was historically limited (Flores-Villela et al., 2004), recent studies have significantly expanded our understanding of the region’s amphibians (see Castro-Bastidas, 2024). With regard to landscape modification, agricultural expansion now covers over 50% of the state’s territory (INEGI, 2017), contributing to habitat alteration and increased environmental stress. Although the concentration of anomalies may be partially influenced by sampling bias, the observed patterns are likely driven by a combination of intensified land use and increased localized monitoring efforts. Therefore, further studies are needed to disentangle the relative impacts of ecological stressors from those of sampling effort.

Other states with notable reports include Michoacán, Veracruz, and Estado de México, regions characterized by diverse amphibian communities and varying environmental pressures. Differences in reporting frequency could also be influenced by the uneven distribution of research efforts. The high frequency of anomalies in specific areas underscores the need for systematic studies to determine whether these patterns result from localized environmental pressures, such as pollution or habitat degradation, or from broader trends affecting amphibian vulnerability, such as climate change, emerging infectious diseases, and invasive species (Lips et al., 2006; Stuart et al., 2004). Future research should aim to standardize methodologies across regions to improve comparability and deepen our understanding of the factors driving these anomalies.

### Challenges and recommendations for documenting amphibian anomalies in Mexico

The study of amphibian anomalies in Mexico faces several challenges, including incomplete records, inconsistent methodologies, and small sample sizes. Some species are more frequently sampled, while others are underrepresented, which can affect the identification of patterns. Additionally, many reports are incidental and lack specific details, such as the exact nature of the anomaly, the affected body parts, or the environmental context in which the anomaly was observed. This lack of information hinders the identification of clear patterns and underlying causes. Furthermore, research efforts are unevenly distributed, with certain regions and species receiving disproportionate attention while others remain largely neglected (Flores-Villela et al., 2004). These issues are compounded by the limited number of herpetologists in the country and restricted access to funding, which collectively hinder the development of comprehensive and systematic studies.

To address these issues, we recommend the development of standardized protocols for data collection and reporting based on other systematic studies (e.g., Henle et al., 2017b; Lunde & Johnson, 2012; Meteyer, 2000). These protocols should include precise definitions of anomalies, high-quality photographic documentation, and comprehensive environmental metadata. Collaboration among herpetologists, ecologists, and local communities is essential to expand the research scope and improve data quality (e.g., Forti et al., 2022). Additionally, prioritizing genetic and toxicological analyses can elucidate the mechanisms behind these anomalies. Finally, promoting citizen science initiatives and engaging local communities in monitoring efforts are vital for increasing the geographic scope and volume of data. These approaches will foster a more inclusive and effective framework for understanding and mitigating amphibian anomalies in Mexico.

## Conclusion

This study provides new records of morphological anomalies in amphibians from the Sierra Madre Occidental and synthesizes available data across Mexico, offering the first nationwide estimate of anomaly frequency. The identification of dyscoria as a previously undocumented condition highlights the need for continued monitoring. Our findings reveal substantial inconsistencies in terminology, which limit comparability, emphasizing the necessity of a standardized classification framework. Although the current evidence supports this, further comparative studies using standardized protocols are needed to validate these findings across broader spatial and taxonomic scales (e.g., at the continent or global level). Establishing such a framework would enhance the utility of anomaly reports as early indicators of environmental degradation, contributing to a more effective assessment of ecological health in amphibian populations.

Despite these contributions, our findings should be interpreted with caution due to key limitations. The lack of standardized reporting in many studies, small sample sizes in fieldwork, and the absence of direct environmental or genetic analyses limit the ability to infer causal relationships. Future studies should prioritize broader and more systematic sampling, integration of toxicological and molecular tools, and the development of national reporting guidelines to better understand the drivers of amphibian anomalies in Mexico.

## Data Availability

The datasets generated during and/or analyzed during the current study are available in the Zenodo repository, 10.5281/zenodo.15067177.

## Appendix

**Table 3:** Types of morphological and chromatic anomalies documented in amphibian species from Mexico. The terminology used here reflects that reported by the original authors. A standardized terminology following Henle et al. (2017b) is available in the Supplementary Material

Species	Type	Source
*Agalychnis dacnicolor*	Amely, pelvic malformations	Morales-Lugo et al. (2022)
*Agalychnis taylori*	Black eyed	Hernández-Vázquez et al. (2023)
*Ambystoma amblycephalum*	Piebaldism	Fraustros-Sandoval et al. (2024)
*Ambystoma flavipiperatum*	Partial leucism	Cortés-Vázquez et al. (2016)
*Ambystoma mexicanum*	Polydactyly	Recuero et al. (2010)
*Ambystoma ordinarium*	Partial missing gill, ectrodactyly, micromely, incomplete tail, additional number of gills, syndactyly, missing gills, brachydactyly, ectromely, polydactyly, regenerated tail, polymely, microdactyly, gill fusion	Soto-Rojas et al. (2017)
*Ambystoma rivulare*	Ectrodactyly, syndactyly, polydactyly, brachydactyly, hypoplasia, polymely	Sánchez-Manjarrez et al. (2022)
*Ambystoma velasci*	Brachydactyly, ectrodactyly, polydactyly	Cruz-Pérez et al. (2009)
	Amelanism	Del Moral-Flores et al. (2022)
*Aquiloeurycea cafetalera*	Microphthalmia	Díaz-García et al. (2019)
*Aquiloeurycea cephalica*	Ocular deformity	Cante-Bazán and Ramírez-Bautista (2023)
*Bolitoglossa platydactyla*	Anophthalmia	Venerozo-Tlazalo et al. (2022)
*Bolitoglossa rufescens*	Melanism	Vásquez-Cruz et al. (2020)
*Craugastor rhodopis*	Femoral ectromely	Díaz-García et al. (2019)
*Dryophytes arenicolor*	Polyphalangy	Monroy-Vilchis et al. (2015)
	Humeral ectromely, brachygnathia, deformity due to parasites, brachydactyly, ectrodactyly, microphthalmia	Castro-Bastidas et al. (2024)
	Brachydactyly	This study
*Dryophytes eximius*	Ectromely	Olvera-Mendoza et al. (2023)
*Hypopachus variolosus*	Radio-ulna ectromely	Reyes-Servín and Díaz-García (2023)
	Albinism	Ruiz-Álvarez et al. (2024)
*Incilius marmoreus*	Ectrodactyly	Rangel-Torres et al. (2023)
*Incilius mccoyi*	Limb deformity	This study
	Limb protrusions	This study
*Incilius occidentalis*	Anophthalmia	Castro-Torreblanca and Blancas-Calva (2021)
*Isthmura bellii*	Polydactyly	Peralta-Robles et al. (2023)
*Leptodactylus fragilis*	Protrusions on the body	Mendoza-Velázquez et al. (2022)
*Lithobates forreri*	Brachydactyly, ectrodactyly, hemimely, brachygnathia, abnormal iris coloration, incomplete interdigital membrane	Monroy-Vilchis et al. (2015)
	Microphthalmia	Castro-Bastidas et al. (2022b)
	Cutaneous fusion	Lynch (1965)
*Lithobates maculatus*	Ocular abnormality	Hernández-Vázquez et al. (2023)
*Lithobates magnaocularis*	Femoral ectromely	This study
	Radio-ulna ectromely	This study
	Dyscoria, microphthalmia, inferior brachygnathia	This study
*Lithobates neovolcanicus*	Femoral ectromely, polymely	Barragán-Ramírez and Navarrete-Heredia (2011)
*Lithobates spectabilis*	Tail malformation, swollen head, lip malformation, lack of melanophores	Méndez-Tepepa et al. (2023)
*Lithobates zweifeli*	Micromely	Monroy-Vilchis et al. (2015)
*Plectrohyla acanthodes*	Polymely	López-Méndez and Aranda-Coello (2021)
*Pseudoeurycea leprosa*	Anophthalmia	Cante-Bazán and Ramírez-Bautista (2023)
*Rheohyla miotympanum*	Skin strap	Venerozo-Tlazalo et al. (2022)
	Brachydactyly, ectrodactyly	Carmona-Zamora et al. (2020)
	Partial melanism	Aguilar-López et al. (2017)
*Rhinella horribilis*	Agenesis, hypoplasia	Domínguez-Moreno et al. (2018)
	Ocular deformity	Castro-Bastidas et al. (2022a)
	Anophthalmia	This study
*Smilisca baudinii*	Anophthalmia	Hernández-Jáuregui et al. (2021)
	Anophthalmia	Aréchaga-Ocampo and Roa-Mata (2022)
	Anophthalmia, prognathism, urostylus deviation	Hernández-Vázquez et al. (2023)
	Axantism	Vásquez-Cruz et al. (2020)
	Axantism	Hernández-Vázquez et al. (2023)
*Smilisca fodiens*	Hypoplasia	Loc-Barragán (2016)
*Triprion spinosus*	Black-eyed	Cerón-de la Luz and Vásquez-Cruz (2021)

**Table 4:** Definitions of chromatic and morphological anomalies in amphibians of Mexico. Definitions are presented according to Henle et al. (2017b). Asterisk (*) symbol indicates that the definition was adapted from Ross and Plummer (2022)

Type	Definition
Chromatic anomalies	
Albinism	Partial or complete absence of pigmentation in the integument, giving the individual a whitish, yellowish to golden, or pinkish to reddish appearance. Includes individuals with transparent skin if the entire body is affected. Cases of both complete and partial albinism have been reported in Mexico. The axolotl (*Ambystoma mexicanum*) exhibits wild-type albino phenotypes
Amelanism	Complete absence of melanin or melanophores
Axanthism	Absence of xanthophores or carotenoid vesicles in the xanthophores. If the normal phenotype is green, these individuals appear blue
Black-eyed	Refers to a black-colored iris. In some species this is the normal phenotype. When unilateral, it is a subcategory of heterochromia
Leucism	Near-total absence of pigmentation in the integument but with pigmented eyes. Often confused with albinism in the literature. The axolotl (*A. mexicanum*) also shows wild-type leucistic phenotypes
Melanism	Complete black coloration of the body or parts of the body. Some species exhibit a completely black coloration as the normal phenotype
Piebaldism	Congenital genetic condition characterized by hypopigmented areas of skin alongside normally pigmented areas. Caused by a genetic mutation affecting pigment cell production or migration during embryonic development
Morphological anomalies	
Amely	Complete absence of one or more limbs; a specific form of ectromely
Anophthalmia	Absence of one or both eyes, resulting in blindness
Apody	Partial or complete absence of the foot (or hand); a specific form of ectromely
Brachydactyly	Abnormally short fingers, caused by a reduced number of phalanges (hypophalangy) or shorter phalanges (brachyphalangy), or both
Brachygnathia	Abnormal shortening of the jaw (micrognathia or brachygnathia). It is generally used as a subcategory or synonym of mandibular hypoplasia. Cases in Mexico differ structurally from typical mandibular hypoplasia
Deformity	Alteration of an organ or structure that was originally formed correctly
Dyscoria*	Irregular shape of the iris
Ectrodactyly	Partial or complete absence of one or more fingers
Ectromely	Partial or complete absence of a limb. Reported cases in Mexico include femur, humerus, and radius-ulna ectromely
Gill anomaly	Abnormal development of gills
Hypoplasia	Underdeveloped or completely absent lower jaw. Cases in Mexico differ from brachygnathia as they refer to entirely absent jaws
Kinking	Abnormal deviation in the spine or tail
Micromely	Proportionally smaller limbs with all elements present
Microphthalmia	Abnormally small eyes
Myiasis	Lesions or malformations caused by parasitic fly larvae laying eggs in living individuals
Ocular reabsorption	Opercular fold abnormally wide, covering the eye. Known as “skin strap” in Mexican literature
Parotoid gland agenesis	Congenital absence of one or both parotoid glands
Polydactyly	Duplication of one or more fingers or parts thereof
Polymely	Duplication of a complete limb or parts thereof
Polyphalangy	Additional bone inserted into a finger without splitting the digit (linear arrangement)
Prognathism	Abnormally large lower jaw. One case reported in Mexico described an individual with an extended lower jaw
Protrusions on the body	Possible fluid accumulations, tumors, tissue masses, edema, blisters, or parasites. May result from developmental errors or deformities
Skin fusion	Abnormal fusion of skin in certain areas. Reported cases in Mexico involve anomalies in the interdigital membrane
Syndactyly	Partial or complete fusion of two or more fingers due to soft tissue fusion or bone fusion (synphalangy)

## Declarations

### Declarations

All authors have read, understood, and have complied as applicable with the statement on “Ethical responsibilities of Authors” as found in the Instructions for Authors.
